# 胸腺恶性肿瘤预后的标准评估方法

**DOI:** 10.3779/j.issn.1009-3419.2014.02.10

**Published:** 2014-02-20

**Authors:** James Huang, Frank C. Detterbeck, Zuoheng Wang, Patrick J. Loehrer, 北京大学肿瘤医院 付浩

**Affiliations:** 1 Division of Thoracic Surgery, Department of Surgery, Yale University School of Medicine and Department of Biostatistics, School of Epidemiology and Public Health, Yale University, New Haven, Connecticut; 2 Thoracic Service, Memorial Sloan-Kettering Cancer Center, New York City, New York; 3 HH Gregg Professor of Oncology, Division of Medical Oncology, Department of Internal Medicine, Indiana Universitychool of Medicine, Indianapolis, Indiana

胸腺恶性肿瘤相对少见，发病率约2.5-3.2/100万^[1, 2]^，病例也分散在各个医疗中心。现有文献几乎都是单中心的回顾性研究，为获得足够的病例，研究时间往往跨度几十年，报道结果的方式和使用的定义也各不相同，不同的研究结果很难比较。加之胸腺恶性肿瘤的进展、复发模式及致死原因等都具有其一定的特殊性，如何报道其结局显得十分重要。由于胸腺恶性肿瘤患者数量相对有限，从现有的经验中收集信息较为困难，而且存在过度描述和错误结论的风险。因此，专业胸腺肿瘤学术组织（International Thymic Malignacy Interest Group, ITMIG）对临床试验结果的报告制定了一套标准，已被该组织承担的合作项目采用。该标准的广泛应用将增强不同研究结果的可比性。只有采用统一的定义和规范的结果报告，胸腺肿瘤研究才能取得重要进展。2010年以前由外科医生、肿瘤内科医生和统计学家组成的小组共同回顾已有文献使用的评估方法形成初步的建议，再经过一个扩展小组审查，并最终将提炼的建议分发给所有ITMIG成员进行进一步讨论。最后在2010年5月6日的纽约年会上得到ITMIG的认可并被采用。本文就ITMIG标准作一综述。

## 1 分期的描述

目前，关于胸腺恶性肿瘤还没有国际抗癌联盟（International Union Against Cancer, UICC）和美国癌症联合委员会（American Joint Committeeon Cancer, AJCC）的官方分期，但已经存在一些分期系统^[3]^，包括Masaoka分期^[4]^、Masaoka-Koga分期^[5]^、GETT分期^[6]^和TNM分期^[7]^。大多数中心和发表的文章都采用Masaoka分期系统，但自1995年后更多学者开始使用Koga改良分期系统（Masaoka-Koga分期），见表 1。也是ITMIG目前建议使用的分期。Koga改良分期与以往的Masaoka分期的不同之处在于，对于包膜侵犯但未侵透，Masaoka分期归为IIb期，而Masaoka-Koga分期归为I期。这是因为大多数病理学家认为包膜部分侵犯没有意义，而且生存数据似乎也证实这一点^[5, 8, 9]^。此外，该分期系统关于包膜完整胸腺瘤和侵袭性胸腺瘤的定义与ITMIG是一致的。另外一个不同之处是，与周围结构粘连或肉眼侵犯但未侵透纵隔胸膜或心包，Masaoka-Koga归为Ⅱb期，而Masaoka未给出明确的定义。尽管Masaoka-Koga分期系统得到广泛应用，但是也有很多定义不明确之处，详见其它有关分期的介绍。ITMIG和IASLC将承担对该分期系统的评价和统计验证，并提出可能的替代分期方法，这就要求收集的数据不止Masaoka-Koga分期，ITMIG数据库对此会单独说明。

**1:** Masaoka-Koga分期系统 Masaoka-Koga staging system

分期	定义
Ⅰ	肉眼和镜下肿物包膜完整
Ⅱa	镜下侵透包膜
Ⅱb	肉眼侵犯胸腺或周围脂肪组织，或与纵隔胸膜、心包粘连，但镜下没有侵犯
Ⅲ	肉眼侵犯邻近器官（如心包，大血管或肺等）
Ⅳa	胸膜或心包转移
Ⅳb	淋巴或血行转移
来自Pathol Int. 注：本表已获得版权所有者© 2011 by the International Association for the Study of Lung Cancer复制许可。	

肿瘤分期的焦点一直都是病理分期。然而，在实际应用中临床分期更为重要，因为手术切除在治疗中往往不是第一步。遗憾的是，肿瘤的相关特征和分期检查的可靠性在临床分期中还没有明确定义。预计这个问题在将来发表的文章中会详细说明。在明确这些定义之前，建议作者基于自己的最佳判断，根据Masaoka-Koga分期系统进行分期。强烈鼓励作者不仅要报道病理分期，还要报道临床分期。

## 2 生存的描述

一个标准的预后评估指标是总生存，它是一个硬性的容易验证的指标，应该在任何胸腺瘤的临床研究结果中予以报道。对许多其他肿瘤而言总生存是一个评估肿瘤预后的良好指标，因为多数肿瘤一旦复发其生存期一般很短，且大多死于与原发肿瘤相关的原因。然而，用总生存来评估胸腺瘤预后存在一定问题，因为胸腺瘤非常特别，尤其是许多I期和II期的胸腺瘤患者，因生存时间长常死于其他原因而非胸腺瘤本身，即使是肿瘤复发患者也能存活很多年，（图 1A，图 1B）。因此，除了总生存之外需要更多特异性指标来评估生存。这些特异性指标通常需考虑特异死亡原因或复发类型，或二者均考虑。死亡原因应准确定义避免将死亡过度归因于癌症，这一问题已受到广泛关注^[10-12]^，这可能更适用于胸腺瘤。然而，在现有胸腺瘤相关文献中普遍存在下列问题，什么是肿瘤相关死亡原因？何谓无疾病生存、无复发生存、肿瘤相关生存及无进展生存等。每个定义都代表不同的结局事件，见表 2。由于这些事件在胸腺瘤的发生率很高，这些定义对胸腺瘤的预后描述非常重要。此外，约15%-20%的胸腺瘤患者已患或可能患第二原发肿瘤^[3, 13, 14]^。图 2举例说明了Ⅲ期胸腺瘤预后评估使用的诸多不同定义。

**1:**
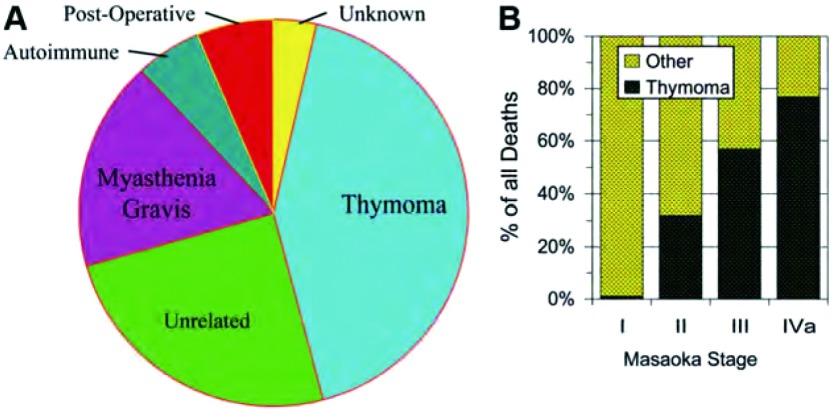
胸腺瘤患者术后所有死因（A）和分期相关死因（B）。结果来自1980年至2009年样本量大于等于100例的研究结果的平均值。 Overall cause (A) and stage-specific (B) cause of death after resection of patients with thymoma. Results are an average of studies from 1980 to 2009 of100 patients reporting this data3.

**2:** 生存评估方法 Survival measures

方法	观察终点	删失观察^a^	包括患者
总生存	死亡，任何死因		全部^b^
疾病相关生存	死于胸腺恶性肿瘤，重症肌无力^c^，治疗	非相关死亡^d^，死因不明	全部^b^
疾病特异生存	死于胸腺恶性肿瘤，重症肌无力^c^	非相关死亡^d^，死因不明	全部^b^
病因特异生存	死于胸腺恶性肿瘤	非相关死亡^d^，死因不明	全部^b^
肿瘤特异生存	死于任何肿瘤	非相关死亡^d^，死因不明	全部^b^
无疾病生存^e^	死亡，复发	复发状态不明	R0切除/完全缓解
无复发生存	复发	非复发死亡；复发状态不明	R0切除/完全缓解
无进展生存	死亡；胸腺恶性肿瘤进展	胸腺恶性肿瘤状态不明	R1, 2切除/部分缓解，进展
进展时间	胸腺恶性肿瘤进展	无进展死亡，胸腺恶性肿瘤状态不明	R1, 2切除/部分缓解，进展
^a^所有分类中都包括失访或研究结束未出现终点事件的患者。^b^所有患者或仅限于某组患者（如，R0切除患者）。^c^重症肌无力或其他胸腺瘤相关疾病（如，红细胞再生障碍或低丙种球蛋白血症）。^d^根据相关事件定义^e^无疾病生存常与无复发生存意义相同，但有时无疾病生存终点事件还包括重症肌无力。 注：本表已获得版权所有者© 2011 by the International Association for the Study of Lung Cancer复制许可。			

**2:**
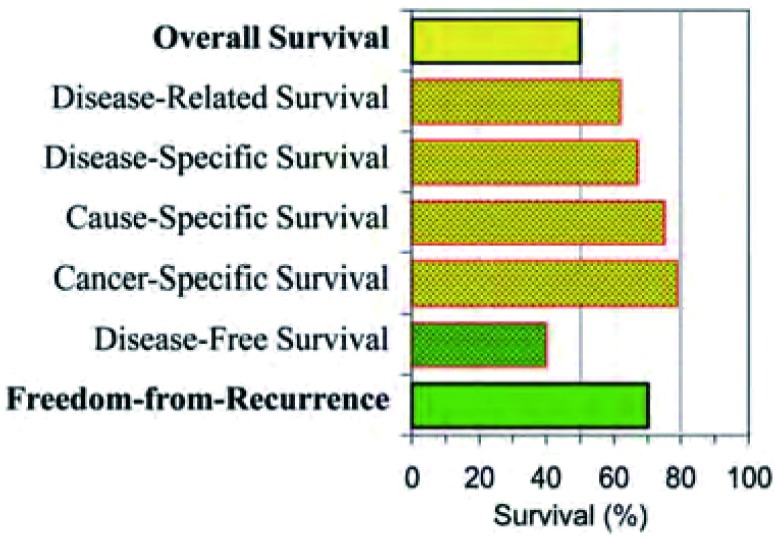
Ⅲ期胸腺瘤术后患者的10年特异性生存，从总生存、死因、复发和第二原发肿瘤发生的相关数据评估^[3]^。 Specific outcomes for a stage Ⅲ resected thymoma at 10 years, estimated from data regarding overall survival, cause of death, incidence of recurrence, and incidence of other cancers^[3]^.

描述特定结局事件的精算结局曲线（比如单独计算无局部复发或特定病因的生存曲线）通常具有误导性，产生过于乐观的结果^[15]^。原因在于精算方法在统计学上要求各个事件之间相互独立，事实上并非如此，比如局部复发时间和远处复发时间很可能是相互关联的^[15]^。根据结局事件的关联程度不同，采用精算法预测一种结局事件的发生率容易比实际发生率低估30%-50%^[15]^。因此，最好的办法是先大体上分析死亡或者其他事件，然后再分析死亡或者发生事件的原因风险比例，从而避免不同原因之间的竞争风险^[15]^。因此，建议报道复发类型和死亡原因的风险比例。

ITMIG推荐的评估胸腺肿瘤疗效的方法是计算其发生在任何部位的复发率，控制重症肌无力等相关疾病的能力应单独考虑。此外，受其他因素的影响死亡原因仅是次优的评估指标，当然不混杂复发的死亡是最理想的评估指标。因此，建议以无复发生存作为根治性切除术或放疗后完全缓解患者预后的最佳评估指标。对于未根治的患者，疾病进展时间是最好的评估指标，疾病进展时间也可以用于R1切除的患者，因为其存在残余病灶，见表 3。不难看出，这两个评估指标的终点是一样的，都是疾病复发，但选择哪个术语取决于治疗后是否仍然存在肿瘤。姑息性治疗的患者选择疾病进展时间，是肿瘤内科医生容易接受的评估指标。根治患者选择无复发生存而非复发时间是因为前者强调阳性结局的可能，而后者给人印象是复发仅为一种时间形式。

**3:** 预后评估方法推荐 Recommended outcome measures

方法	病例组	起点
总生存	所有患者	诊断时间
无复发生存	R0切除或经放疗/放疗后影像学CR的患者	治疗完成时间
进展时间	R1，2切除，影像学SD或PR的患者	治疗完成时间
CR：完全缓解；PR：部分缓解；SD：疾病稳定。 注：本表已获得版权所有者© 2011 by the International Association for the Study of Lung Cancer复制许可。		

由于胸腺瘤的惰性生物学行为和复发后能长期存活的事实，建议对于总生存在报道5年生存后还需要报道10年生存。但对于胸腺癌仍建议采用5年生存较为合适。胸腺瘤完全切除后平均复发时间约为5年（3年-7年）^[13, 16-21]^。一项研究表明，根据分期的不同复发时间也有所差别，I期胸腺瘤患者平均复发时间为10年，而II期至IV期胸腺患者平均复发时间为3年^[22]^，说明肿瘤生物学行为越惰性，复发时间越长。鉴于这些原因，建议对于无复发生存使用5年生存较为合适，尽管对于I期肿瘤，它可能好于5年和10年生存率。

人们经常忽略这样一个概念，因为患者可能会失访或没有达到研究随访时间，生存曲线提供的只是一个生存估计，其变异程度取决于样本量、随访时间、研究时间和中位生存，如果样本量少于50例，则变异度会很大，而且在短期研究中表现会更明显，见图 3A、B。对于少见疾病，大样本是不容易获得，因此对获得数据局限性的评估是很重要，应该提供生存估计的置信区间。建议在所有研究中报道中位随访时间（把患者入组到出现感兴趣事件、研究结束或失访的时间，作为整个研究的中位数）。

**3:**
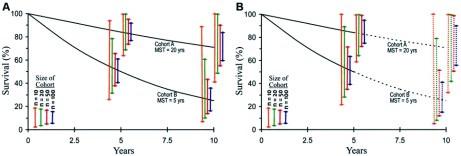
队列样本量不同，持续10年的研究（A）和持续5年的研究（B）在精确估算生存上存在差异。竖线代表根据标准的指数生存模型（研究过程中患者是固定的并且零失访）估计出5年和10年生存的95%置信区间。 Variance in actuarial survival estimates by size of cohort for (A) a 10-year study duration and (B) a 5-year study duration. The vertical bars are 95% confidence intervals for the survival estimate at 5 and 10 years, based on a standard model of exponentially decreasing survival, a constant rate of accrual of patients during the course of the study until study termination, and no loss to follow-up. MST, median survival time.

## 3 手术切除完全性的描述

多因素分析表明，完整手术切除是一个主要的预后因素^[3, 23]^。然而，如何处理标本和如何报道切缘在不同的研究中存在巨大的差异，因为解剖特征和胸腺周围常常有疏松结缔组织会误导分期。对于胸腺肿瘤切除标本的处理流程和病理报告ITMIG已另文报道，旨在减小这种误导分期的风险。简而言之，在切除的过程中需标记关注区域，以减少相关区域组织的破坏，和使外科医生和病理科医生能清楚标本的方向以便更好的和病理科医生交流。病理科工作人员应小心处理大体标本，对关注区域分别切片。切缘阳性提示肿瘤侵犯达到切除标本的墨水标记的表面。如果肿瘤暴露在纵隔胸膜和心包表面，但是与正常体腔有分界（无粘连表现），不能归为切缘阳性。在胸腺瘤切除过程中存在一个问题，就是胸腺周围的疏松结缔组织很容易被破坏，从而导致肿瘤暴露，事实上肿瘤并未达到切缘表面。如果确定肿瘤暴露区域不是关注部位而且确定在处理过程中遭到破坏，那么这些区域不应该被归为切缘阳性。

## 4 复发定义

这一术语适合于所有疾病已被根治或潜在根治如R0切除术后者或根治性放疗后的完全缓解者。须注意的是，当临床高度怀疑（或证实）复发时，就定义为复发而勿需影像或活检等特异性的检查加以证实。因为带瘤生存有时仅根据复查就能判断，要求组织活检证实或需要治疗了才定义为复发，会低估复发率。复发时间应记录为首次高度临床怀疑的时间，即使以后有检查（影像或活检）进一步证实临床诊断。当高度怀疑复发时，建议尽可能取活检。少数情况下，可能高度怀疑复发的诊断在后来被证实为错误的，如活检证实或肿瘤自行消退，对于这类病例应取消复发的诊断。但复发的首诊时间不应回顾性制定，这意味着不能将复发首诊时间设置为临床怀疑诊断之前，即使回顾审阅影像片时发现原来有遗漏的异常，而且该异常在后来被证实为复发。影像随访检查的频率可能会影响首次怀疑复发的时间。我们建议在手术切除后5年内，每年复查胸部CT，之后改为每年行胸片检查，直到11年。切除的Ⅲ期或IV期胸腺瘤、胸腺癌、不完整切除，或其他高风险肿瘤，建议3年内每6个月做一次胸部CT。术后在炎症消退后（如：术后4周-12周）做一次基线检查对于以后比较是非常有用的。MRI在图像分辨率或降低放射剂量上优于CT。PET不作为常规检查手段，但在某些情况可能从中获益，如临床或影像怀疑有复发者。有人提出频率更高的随访方案，如终生每年做CT检查，但这在世界不同的医疗机构并没有得到广泛认可。

### 4.1 局部复发的定义

局部复发应定义为胸腺床部位，如前纵隔或正常胸腺或胸腺瘤邻近组织中出现的新病灶（表 4）。包括原发肿瘤邻近的受累淋巴结、胸膜或心包肿物。也包括出现在之前切除的胸膜转移（Ⅳa期）的部位的新病灶，但应额外注明“转移部位复发”。最后，病灶出现在邻近胸腺上级的下颈部时，应定义为局部复发。

**4:** 复发的定义（R0切除或影像学完全缓解） Definitions of recurrence (after R0 resection or radiographic complete response)

局部复发——前纵隔
胸腺或切除胸腺瘤床处肿瘤复发
包括紧邻胸腺或胸腺瘤的心包，胸膜或肺的肿瘤复发
紧邻胸腺或切除胸腺瘤的淋巴结复发（包括紧邻胸腺上极的颈部淋巴结）
原淋巴结转移部位的复发（Ⅳa期）——需特殊注明
区域复发——非邻近胸腺或切除胸腺瘤的胸腔内复发
壁胸膜结节
心包结节
脏层胸膜结节
非邻近胸腺或胸腺恶性肿瘤的纵隔淋巴结
远处复发
胸腔外复发
肺内结节（结节与脏层胸膜之间有正常肺组织）
注：本表已获得版权所有者© 2011 by the International Association for the Study of Lung Cancer复制许可。

### 4.2 区域复发的定义

区域复发定义为发生在胸腔内的复发肿瘤，但不与胸腺或原胸腺肿瘤的部分紧邻，这包括胸膜壁层或脏层和心包结节（表 4），但不包括原发肿瘤床的部位。包括叶间裂在内的邻近的胸膜结节定义为脏层胸膜结节。区域复发也包括与胸腺或胸腺瘤不相邻的淋巴结，如食管周围或更远的颈部淋巴结。

### 4.3 远处复发的定义

远处复发包括胸腔外和下颈部组织，腹膜腔或腹膜后结节，但不包括起源于胸腔内结节侵透膈肌进入腹膜腔或腹膜后的部分。建议远处复发也应该包括有明确界限的肺内结节，即病灶存在影像学或肉眼上与脏层胸膜可分辨的界限。这基于一个推理，脏层胸膜下结节的播散途径是通过胸膜腔，而肺实质内结节是通过血行途径。但是，没有数据证实这一推理，也没有数据表明这样区分有临床意义。为了前瞻性的研究，仅为肺内的远处复发结节应单独记录，与胸腔外复发区分开。

## 5 化疗和放疗疗效的描述

化疗和放疗常作为手术前的诱导治疗，也作为不可手术切除患者的根治性治疗，或者姑息性治疗手段。疗效评估的标准方法是R ECIST标准（1.1版），用CT测量肿瘤一维径线^[24]^。尽管该标准可能适合于大多数肿瘤，但胸腺肿瘤的解剖因素，包括大小、部位、边界不规则和与周围结构的紧密关系，很难对其做连续性的测量。无论是手测或电子卡尺测量，在观察者内和观察者之间都存在差异，尤其对边界不规则或边界模糊的肿瘤^[25, 26]^。因此，我们建议肿瘤评估应由单人操作，理想的人选是有测量经验的放射科医生^[25]^。胸腺肿瘤治疗反应还可能表现为囊性变、中心坏死和密度改变，传统测量最大径的方法不能涵盖这些内容。另一个问题是，对于富含淋巴细胞型胸腺瘤化疗或强的松治疗就可以看到明显的反应，但影响的可能是正常淋巴细胞，而非肿瘤细胞。其它复杂因素包括胸腺肿瘤累及胸膜的倾向，这对RECIST标准提出了很大挑战。尽管RECIST标准将胸膜结节排除在考虑之外，但由于胸膜结节对胸腺瘤和间皮瘤都十分重要，应该包括在测量之内^[27]^。相比一维测量方法评估治疗反应，肿瘤体积的测量可能更客观、准确和稳定。但这些方法需要进一步研究和证实。

在新的评效方法出来之前，建议遵循新版RECIST标准（1.1版）^[24]^。所有可测量的病灶每个器官最多选取2个，病灶总数不超过5个，代表所有受累器官，把它们作为靶病灶，进行基线测量和记录。靶病灶应根据它们的大小（如最大径线LD）和适合反复准确测量来选取。计算它们的最大径线之和，作为评估肿瘤治疗反应的参考^[28]^。这个标准的例外是胸膜病灶，因为相比沿胸膜延伸的长轴，胸膜病灶的厚度能更明确且能被稳定的测量^[27]^。对于广泛的胸膜受累病灶，在胸部CT上应对病灶垂直于胸壁或纵隔的厚度，在两个不同位置三个层面上进行测量^[27]^。将6个测量结果之和规定为胸膜一维测量值。把胸膜一维测量之和与非胸膜靶向病灶之和规定为总的最大径基线，与之后的肿瘤治疗反应或进展相比较^[24, 27]^。

疾病进展应根据1.1版R ECIST标准定义^[24]^。当然，疾病进展的诊断时间也受评估频率的影响。我们建议，对于Ⅲ期或Ⅳ期胸腺瘤、胸腺癌，不完整切除，或高风险肿瘤，3年内每6个月行一次影像学检查^[21]^。早期肿瘤按照复发定义部分的描述，每年行影像学检查。

治疗反应也可以通过化疗或放疗的肿瘤组织来评估。炎症、坏死和纤维化通常被视为混有残余肿瘤，治疗反应的程度可以根据镜下残余肿瘤细胞的比例来定量，以10%递增。当确定整个标本没有残余肿瘤细胞时，定义为完全病理缓解。切片的数量对该诊断是非常重要的。建议在肿瘤直径上每厘米要有一张切片。

## 6 多因素分析

统计软件的应用很容易进行多因素分析来研究潜在的预后因素。然而，作者往往对统计学不是很了解，导致结论常被夸大。基本上目前所有的预后因素研究都是探索性的，是所谓的Ⅰ期预后因素研究。这样探索是没有问题的，但是外部验证是必须的^[29]^。例如，当应用步进式回归分析时，可能会出现不同预测因素，这取决于使用的正演模型或向后模型和引入变量的顺序。最终模型中的回归系数通常会被高估，那么在这些研究中的*P*值实际上是无效的（因为用于定义变量的数据和用于评估预后的数据是一样的）^[29]^。在不调整其他因素的情况下，这同样适用于有或没有预后因素的生存曲线。此外，当在最佳区分能力的基础上选择一个界值来二分连续变量，假阳性预后因素的概率约为40%，尽管*P*值小于0.05^[30]^。自举法可以克服这些问题^[31]^。

在研究潜在预后因素的时候，出现假阴性结果的概率也是很高的，这主要是因为样本量不足。当实际过程中样本量小到不足以发现差异时，很容易得到的结论是一个因素没有独立的预后意义。一般估计需要的最小样本量，可以使用一个在线的多元回归分析工具来计算，见表 5^[32]^。这仅仅被视为一个粗略的估计，因为队列中其他因素（例如预后因素的不均衡分布）也起作用。更详细的分析需要统计学家的参与。

**5:** 多元回归分析所需样本量 Sample size needed for multiple regression analysis

待检验预测指标的数量	检验效能=0.8 效应值^a^			检验效能=0.6 效应值^a^		
	小	中	大	小	中	大
2	478	67	31	308	44	21
3	543	76	36	356	52	25
4	597	84	39	395	58	28
5	643	91	43	429	63	31
6	684	97	46	460	68	33
7	721	103	49	488	72	36
8	755	108	52	513	76	38
9	788	113	54	538	80	40
10	818	118	57	561	84	42
特定数量的预测指标所需最小样本量的估计值，假定检验水准为0.05 Values calculated using an online tool available at: 采用在线工具进行计算(http://www.danielsoper.com/statkb/topic01.aspx.32) ^a^效应值的大小采用常规的取值方法，(f2)值为0.02, 0.15, 0.35分别表示小，中，大 注：本表已获得版权所有者© 2011 by the International Association for the Study of Lung Cancer复制许可。						

因为在少见疾病中样本量是主要问题，例如胸腺瘤，进行多因素分析和对结果进行解释时一定要注意。我们建议，预后因素的多因素研究应请求统计学家的帮助。研究应包括定义选择的分析参数（如检验水准和效能水平）。此外，结果应以正确的观点说明（例如“本研究说明几个因素可能对预后没有重大影响，尽管样本量不足以评估一个小的或中等影响”或“这些因素似乎没有预后意义，但是在该有限的研究中，检测效能仅为0.4”）。理想情况下，每个变量的风险比应报道95%置信区间。大多数情况下，在得出任何正式结论之前，研究结果必须进行外部验证。

一种共同语言对分享来自不同中心的经验是必不可少的，而且胸腺恶性肿瘤发病率低，多中心合作是取得足够样本的关键。ITMIG是对该疾病感兴趣人士组成的组织，致力于提供平台和方法以促进共同研究。本文为ITMIG关于如何报道结果奠定了基础。阐明了如何定义研究终点的细节，避免因为缺乏统一性而产生歧义和无法结合现有数据。这些定义将用于ITMIG合作项目中。我们也希望这将成为胸腺恶性肿瘤一般研究实用指南。本文提出了一些有关临床研究结果分析的统计学问题，因为它们适用于胸腺瘤这样相对少见和惰性的疾病，从小样本研究中得出的结论，其可信度存在局限性。这并不是不鼓励单中心研究，相反，是有助于一般医疗机构保持现有数据的真实性。可以理解，人们常试图从有限的经验中尽可能多的获取信息，但应意识到其价值是有限的，若没有意识到其可信度的限制，则会得出错误的结论。
